# Supplementary material for: Occupational Mortality Matrix: A Tool for Epidemiological Assessment of Work-Related Risk Based on Current Data Sources
Source: Int J Environ Res Public Health. 2022 May 6;19(9):5652. doi: 10.3390/ijerph19095652 (PMC9104125; doi:10.3390/ijerph19095652)
Supplement: Supplementary file 1 [file ijerph-19-05652-s001.zip › ijerph-1654922-supplementary.pdf]

Table S1 Occupational Mortality Matrix (OMM) based on proportional mortality ratios (PMRs) by malignant neoplasm and industrial sector – Males (*).

		Males					
Cause of death	Industrial Sector	N	PMR	N_5	PMR_5	N_{10}	PMR_{10}
All neoplasms	Railways	4353	1.19 (1.16-1.21)	624	0.98 (0.92-1.05)	3729	1.14 (1.12-1.17)
	Manufacture of refined petroleum products	2571	1.14 (1.11-1.17)	689	0.98 (0.93-1.05)	1882	1.17 (1.13-1.21)
	Manufacture of air and spacecraft and related machinery	1532	1.12 (1.07-1.16)	321	0.96 (0.88-1.05)	1211	1.11 (1.07-1.16)
	Manufacture of pharmaceutical preparations	3161	1.12 (1.09-1.15)	940	1.08 (1.03-1.14)	2221	1.10 (1.07-1.14)
	Photographic activities	361	1.10 (1.02-1.19)	215	1.17 (1.06-1.30)	146	1.11 (0.98-1.24)
	Printing	6526	1.10 (1.08-1.12)	2212	1.06 (1.03-1.10)	4314	1.10 (1.07-1.12)
	Land transport	24534	1.10 (1.08-1.11)	7638	1.05 (1.03-1.07)	16896	1.09 (1.08-1.10)
	Manufacture of basic chemicals	14588	1.09 (1.07-1.10)	5390	1.02 (1.00-1.04)	9198	1.11 (1.10-1.13)
	Building of ships and boats	3476	1.09 (1.06-1.12)	1120	0.96 (0.91-1.00)	2356	1.14 (1.11-1.17)
	Electricity, gas, steam and air conditioning supply	8194	1.09 (1.07-1.11)	1007	0.97 (0.92-1.02)	7187	1.05 (1.03-1.07)
	Manufacture of basic metals	21417	1.09 (1.08-1.10)	7484	0.99 (0.97-1.01)	13933	1.13 (1.12-1.15)
	Manufacture of electrical equipment	15899	1.08 (1.06-1.09)	5659	1.03 (1.01-1.05)	10240	1.08 (1.07-1.10)
	Manufacture of machinery, equipment and motor vehicles	75395	1.07 (1.06-1.07)	26603	1.00 (0.99-1.01)	48792	1.10 (1.09-1.10)
	Manufacture of rubber products	4897	1.07 (1.04-1.09)	1540	0.99 (0.95-1.03)	3357	1.08 (1.05-1.10)
	Manufacture of wearing apparel	4044	1.06 (1.03-1.08)	1996	1.09 (1.05-1.13)	2048	1.07 (1.03-1.10)
	Wholesale and retail trade	40036	1.06 (1.05-1.07)	17238	1.04 (1.03-1.05)	22798	1.09 (1.08-1.10)
	Water transport	3405	1.06 (1.04-1.09)	1903	1.07 (1.03-1.11)	1502	1.12 (1.08-1.16)
	Manufacture of jewellery	920	1.05 (1.00-1.10)	362	1.02 (0.94-1.11)	558	1.07 (1.00-1.13)
	Manufacture of paper and paper products	4573	1.04 (1.02-1.06)	1620	0.97 (0.93-1.01)	2953	1.06 (1.03-1.09)
	Fishing	3064	1.04 (1.01-1.07)	1452	1.03 (0.98-1.07)	1612	1.07 (1.03-1.11)
	Manufacture of plastics products	4915	1.04 (1.02-1.07)	1813	1.00 (0.96-1.04)	3102	1.07 (1.04-1.09)
	Manufacture of leather and related products	6247	1.03 (1.01-1.05)	2468	1.01 (0.98-1.04)	3779	1.05 (1.03-1.08)
	Manufacture of wood products	13653	1.03 (1.01-1.04)	5723	1.01 (0.99-1.03)	7930	1.05 (1.03-1.07)
	Warehousing and storage, rental and leasing activities, travel agency	5137	1.03 (1.01-1.05)	1710	0.95 (0.91-0.99)	3427	1.07 (1.04-1.09)
	Manufacture of textiles	11200	1.02 (1.01-1.04)	4732	0.99 (0.96-1.01)	6468	1.05 (1.03-1.07)
	Financial, Insurance Activities And Business Support Service Activities	27083	1.01 (1.00-1.02)	8070	0.90 (0.89-0.92)	19013	1.06 (1.05-1.08)
Mesothelioma	Building of ships and boats	289	8.15 (7.28-9.13)	70	6.51 (5.16-8.21)	219	8.62 (7.57-9.82)
	Water transport	120	3.27 (2.73-3.90)	53	3.17 (2.42-4.14)	67	3.92 (3.09-4.97)
	Railways	131	3.00 (2.53-3.56)	16	2.65 (1.63-4.31)	115	2.73 (2.27-3.28)
	Manufacture of sugar	19	2.62 (1.68-4.10)	8	2.33 (1.17-4.65)	11	3.36 (1.87-6.03)
	Manufacture of refined petroleum products	62	2.38 (1.86-3.05)	8	1.21 (0.60-2.41)	54	2.62 (2.01-3.42)
	Manufacture of basic metals	449	2.05 (1.87-2.25)	107	1.52 (1.26-1.84)	342	2.27 (2.04-2.52)

	Manufacture of basic chemicals	271	1.78 (1.58-2.01)	86	1.75 (1.42-2.16)	185	1.78 (1.54-2.05)
	Electricity, gas, steam and air conditioning supply	149	1.73 (1.47-2.03)	17	1.73 (1.08-2.78)	132	1.52 (1.28-1.80)
	Manufacture of machinery, equipment and motor vehicles	1301	1.70 (1.60-1.79)	373	1.54 (1.39-1.71)	928	1.77 (1.65-1.89)
	Manufacture of rubber products	89	1.69 (1.37-2.08)	19	1.29 (0.82-2.02)	70	1.78 (1.41-2.25)
	Manufacture of other products n.e.c.	122	1.68 (1.41-2.01)	64	2.05 (1.61-2.62)	58	1.60 (1.24-2.07)
	Manufacture of electrical equipment	259	1.52 (1.35-1.72)	83	1.60 (1.29-1.98)	176	1.47 (1.27-1.70)
	Warehousing and storage, rental and leasing activities, travel agency	86	1.50 (1.21-1.85)	28	1.62 (1.12-2.35)	58	1.43 (1.10-1.85)
	Manufacture of concrete articles, cement and plaster	182	1.42 (1.23-1.65)	55	1.28 (0.98-1.67)	127	1.51 (1.27-1.80)
	Land transport	365	1.41 (1.27-1.56)	58	0.82 (0.63-1.06)	307	1.58 (1.41-1.77)
	Manufacture of plastics products	71	1.29 (1.03-1.63)	22	1.26 (0.83-1.91)	49	1.32 (1.00-1.75)
Larynx	Fishing	68	1.41 (1.11-1.78)	27	1.07 (0.73-1.56)	41	1.70 (1.25-2.31)
	Construction	1845	1.34 (1.28-1.41)	999	1.32 (1.24-1.40)	846	1.38 (1.29-1.48)
	Warehousing and storage, rental and leasing activities, travel agency	101	1.24 (1.02-1.51)	31	0.95 (0.67-1.35)	70	1.42 (1.13-1.80)
Trachea, bronchus and lung	Fishing	955	1.22 (1.15-1.29)	435	1.18 (1.08-1.29)	520	1.28 (1.18-1.39)
	Water transport	1000	1.20 (1.14-1.28)	551	1.22 (1.13-1.32)	449	1.27 (1.16-1.38)
	Land transport	7073	1.19 (1.16-1.21)	2189	1.15 (1.10-1.19)	4884	1.18 (1.15-1.21)
	Manufacture of refined petroleum products	673	1.16 (1.08-1.24)	180	1.03 (0.89-1.18)	493	1.18 (1.08-1.28)
	Building of ships and boats	954	1.15 (1.08-1.22)	278	0.94 (0.84-1.05)	676	1.23 (1.15-1.32)
	Manufacture of basic metals	5916	1.14 (1.12-1.17)	1883	0.97 (0.93-1.02)	4033	1.22 (1.19-1.26)
	Manufacture of pharmaceutical preparations	813	1.13 (1.05-1.20)	248	1.14 (1.02-1.29)	565	1.08 (1.00-1.17)
	Printing	1762	1.13 (1.08-1.18)	579	1.08 (1.00-1.17)	1183	1.12 (1.07-1.19)
	Manufacture of air and spacecraft and related machinery	404	1.12 (1.02-1.23)	80	0.95 (0.77-1.17)	324	1.11 (1.00-1.23)
	Warehousing and storage, rental and leasing activities, travel agency	1464	1.11 (1.05-1.16)	466	0.99 (0.90-1.08)	998	1.16 (1.10-1.23)
	Manufacture of basic chemicals	3841	1.10 (1.06-1.13)	1286	0.96 (0.91-1.01)	2555	1.16 (1.12-1.21)
	Railways	1092	1.10 (1.04-1.17)	150	0.91 (0.78-1.06)	942	1.06 (1.00-1.12)
	Construction	25168	1.09 (1.08-1.11)	12358	1.08 (1.06-1.10)	12810	1.19 (1.17-1.21)
	Manufacture of rubber products	1315	1.08 (1.03-1.14)	378	0.95 (0.86-1.04)	937	1.12 (1.06-1.19)
	Manufacture of basic precious and non-ferrous metals	579	1.08 (1.00-1.17)	220	1.00 (0.88-1.13)	359	1.16 (1.05-1.28)
	Manufacture of ceramic products	1097	1.07 (1.01-1.13)	391	0.97 (0.88-1.06)	706	1.12 (1.05-1.20)
	Wholesale and retail trade	10771	1.07 (1.05-1.09)	4490	1.04 (1.01-1.07)	6281	1.13 (1.10-1.15)
	Manufacture of machinery, equipment and motor vehicles	20085	1.07 (1.06-1.09)	6734	0.97 (0.94-0.99)	13351	1.12 (1.10-1.14)
	Manufacture of electrical equipment	4127	1.06 (1.03-1.09)	1401	0.99 (0.94-1.04)	2726	1.08 (1.04-1.12)
	Mining and quarrying	1172	1.05 (1.00-1.11)	469	0.92 (0.84-1.00)	703	1.19 (1.11-1.27)
	Manufacture of food products	3977	1.03 (1.00-1.06)	1491	0.97 (0.93-1.02)	2486	1.07 (1.03-1.11)
Sinonasal	Manufacture of leather and related products	31	5.04 (3.54-7.19)	15	6.24 (3.74-10.39)	16	4.29 (2.61-7.02)

	Manufacture of wood products	53	4.04 (3.08-5.31)	24	4.42 (2.94-6.63)	29	3.83 (2.65-5.55)
Lymphoid, hematopoietic and related tissue	Manufacture of air and spacecraft and related machinery	131	1.21 (1.03-1.44)	22	0.89 (0.59-1.34)	109	1.25 (1.04-1.51)
	Manufacture of refined petroleum products	210	1.19 (1.04-1.36)	53	1.02 (0.78-1.33)	157	1.23 (1.06-1.43)
	Printing	544	1.17 (1.08-1.28)	202	1.27 (1.11-1.46)	342	1.11 (1.00-1.23)
	Manufacture of rubber products	405	1.16 (1.05-1.27)	121	1.06 (0.89-1.26)	284	1.18 (1.06-1.33)
	Manufacture of basic chemicals	1151	1.12 (1.06-1.19)	421	1.10 (1.00-1.20)	730	1.13 (1.05-1.22)
	Electricity, gas, steam and air conditioning supply	630	1.08 (1.00-1.17)	77	1.01 (0.81-1.26)	553	1.03 (0.95-1.11)
	Manufacture of textiles	897	1.08 (1.01-1.15)	373	1.07 (0.97-1.18)	524	1.11 (1.02-1.21)
	Manufacture of electrical equipment	1220	1.06 (1.01-1.12)	430	1.04 (0.95-1.14)	790	1.07 (1.00-1.15)
	Manufacture of machinery, equipment and motor vehicles	5753	1.06 (1.03-1.08)	2023	1.01 (0.97-1.05)	3730	1.08 (1.05-1.12)
	Financial, Insurance Activities And Business Support Service Activities	2276	1.06 (1.02-1.11)	671	0.94 (0.87-1.01)	1605	1.12 (1.06-1.17)
	Wholesale and retail trade	3084	1.04 (1.01-1.08)	1333	1.04 (0.99-1.10)	1751	1.07 (1.02-1.12)
	Land transport	1822	1.04 (1.00-1.09)	550	0.99 (0.91-1.07)	1272	1.05 (1.00-1.11)
Breast	Manufacture of pharmaceutical preparations	9	2.56 (1.33-4.93)		0.00 (0.00-0.00)	8	3.01 (1.50-6.05)
	Manufacture of plastics products	12	2.07 (1.17-3.65)	3	1.47 (0.47-4.55)	9	2.34 (1.22-4.51)
	Manufacture of food products	26	1.48 (1.00-2.17)	10	1.54 (0.83-2.86)	16	1.43 (0.88-2.34)
	Financial, Insurance Activities And Business Support Service Activities	49	1.44 (1.08-1.92)	15	1.44 (0.86-2.41)	34	1.42 (1.01-2.01)
Colon-Rectum	Photographic activities	50	1.50 (1.15-1.97)	30	1.68 (1.19-2.38)	20	1.45 (0.95-2.21)
	Repairing n.e.c.	41	1.50 (1.11-2.02)	19	1.27 (0.82-1.97)	22	1.91 (1.28-2.86)
	Railways	490	1.31 (1.20-1.42)	69	1.09 (0.87-1.38)	421	1.23 (1.12-1.35)
	Manufacture of jewellery	108	1.22 (1.02-1.47)	41	1.19 (0.88-1.60)	67	1.25 (0.99-1.58)
	Manufacture of refined petroleum products	275	1.18 (1.05-1.32)	72	1.01 (0.80-1.26)	203	1.20 (1.05-1.37)
	Manufacture of pharmaceutical preparations	335	1.14 (1.02-1.26)	95	1.06 (0.87-1.29)	240	1.12 (0.99-1.27)
	Manufacture of electrical equipment	1698	1.13 (1.08-1.18)	581	1.06 (0.98-1.15)	1117	1.14 (1.08-1.21)
	Manufacture of glass and glass products	346	1.13 (1.02-1.25)	112	0.98 (0.81-1.17)	234	1.19 (1.05-1.35)
	Manufacture of leather and related products	666	1.10 (1.02-1.18)	249	1.04 (0.92-1.17)	417	1.13 (1.03-1.24)
	Electricity, gas, steam and air conditioning supply	849	1.09 (1.02-1.17)	114	1.08 (0.90-1.29)	735	1.02 (0.95-1.10)
	Manufacture of rubber products	507	1.09 (1.00-1.19)	144	0.93 (0.79-1.09)	363	1.13 (1.02-1.25)
	Manufacture of machinery, equipment and motor vehicles	7639	1.07 (1.05-1.10)	2710	1.04 (1.00-1.08)	4929	1.08 (1.05-1.11)
	Manufacture of wood products	1429	1.07 (1.02-1.13)	598	1.08 (1.00-1.17)	831	1.08 (1.01-1.15)
	Manufacture of basic metals	2140	1.07 (1.03-1.12)	723	0.96 (0.89-1.03)	1417	1.12 (1.06-1.18)
	Land transport	2397	1.06 (1.02-1.11)	688	0.97 (0.91-1.05)	1709	1.07 (1.02-1.12)
	Wholesale and retail trade	4035	1.05 (1.02-1.09)	1695	1.04 (1.00-1.09)	2340	1.08 (1.04-1.13)
	Financial, Insurance Activities And Business Support Service Activities	2837	1.04 (1.00-1.08)	785	0.90 (0.84-0.97)	2052	1.09 (1.04-1.14)
Liver	Manufacture of wearing apparel	310	1.20 (1.07-1.34)	160	1.27 (1.09-1.48)	150	1.16 (0.99-1.36)

	Electricity, gas, steam and air conditioning supply	587	1.20 (1.11-1.30)	65	0.97 (0.77-1.24)	522	1.19 (1.10-1.30)
	Manufacture of basic metals	1511	1.16 (1.11-1.22)	495	1.01 (0.92-1.10)	1016	1.24 (1.17-1.32)
	Manufacture of paper and paper products	329	1.12 (1.01-1.25)	113	1.03 (0.86-1.23)	216	1.16 (1.02-1.32)
	Printing	444	1.11 (1.02-1.22)	136	0.96 (0.82-1.14)	308	1.18 (1.05-1.31)
	Office administrative and other support activities	2582	1.08 (1.04-1.13)	1775	1.18 (1.12-1.23)	807	1.04 (0.98-1.12)
	Manufacture of basic chemicals	950	1.08 (1.02-1.15)	342	1.01 (0.91-1.12)	608	1.12 (1.04-1.21)
	Land transport	1664	1.08 (1.03-1.13)	515	1.00 (0.92-1.09)	1149	1.11 (1.04-1.17)
	Manufacture of machinery, equipment and motor vehicles	4935	1.03 (1.00-1.06)	1695	0.92 (0.88-0.96)	3240	1.09 (1.05-1.13)
Stomach	Railways	274	1.18 (1.05-1.32)	37	0.99 (0.72-1.36)	237	1.10 (0.97-1.24)
	Manufacture of wood products	986	1.17 (1.10-1.25)	372	1.11 (1.00-1.22)	614	1.24 (1.15-1.34)
	Manufacture of textiles	781	1.14 (1.06-1.22)	297	1.05 (0.94-1.17)	484	1.20 (1.10-1.31)
	Manufacture of wearing apparel	270	1.12 (1.00-1.26)	127	1.18 (0.99-1.40)	143	1.13 (0.96-1.32)
	Manufacture of leather and related products	422	1.10 (1.00-1.21)	172	1.18 (1.02-1.37)	250	1.05 (0.93-1.18)
	Electricity, gas, steam and air conditioning supply	510	1.09 (1.00-1.19)	56	0.92 (0.71-1.19)	454	1.03 (0.94-1.13)
	Manufacture of basic metals	1317	1.07 (1.01-1.12)	430	0.96 (0.88-1.06)	887	1.09 (1.03-1.17)
	Construction	5703	1.04 (1.01-1.07)	2759	1.06 (1.03-1.11)	2944	1.11 (1.07-1.15)
Kidney	Printing	221	1.35 (1.19-1.54)	66	1.21 (0.95-1.54)	155	1.38 (1.18-1.62)
	Fishing	101	1.28 (1.05-1.55)	37	1.03 (0.75-1.42)	64	1.53 (1.20-1.95)
	Manufacture of plastics products	157	1.22 (1.05-1.43)	50	1.07 (0.81-1.41)	107	1.29 (1.07-1.56)
	Warehousing and storage, rental and leasing activities, travel agency	163	1.21 (1.04-1.41)	42	0.91 (0.67-1.23)	121	1.34 (1.13-1.60)
	Manufacture of basic chemicals	431	1.19 (1.08-1.31)	156	1.16 (0.99-1.36)	275	1.18 (1.05-1.33)
	Manufacture of electrical equipment	481	1.19 (1.09-1.30)	163	1.15 (0.99-1.34)	318	1.18 (1.06-1.32)
	Land transport	727	1.18 (1.10-1.27)	232	1.24 (1.09-1.41)	495	1.12 (1.03-1.22)
	Manufacture of machinery, equipment and motor vehicles	2217	1.16 (1.12-1.21)	710	1.04 (0.97-1.12)	1507	1.21 (1.15-1.28)
	Wholesale and retail trade	1179	1.13 (1.07-1.20)	483	1.12 (1.03-1.23)	696	1.17 (1.09-1.27)
	Financial, Insurance Activities And Business Support Service Activities	803	1.07 (1.00-1.15)	225	0.95 (0.83-1.08)	578	1.12 (1.03-1.21)
Bladder	Fishing	155	1.27 (1.08-1.48)	89	1.33 (1.08-1.63)	66	1.21 (0.95-1.54)
	Water transport	164	1.23 (1.05-1.43)	95	1.13 (0.93-1.39)	69	1.41 (1.12-1.78)
	Land transport	1003	1.17 (1.10-1.25)	364	1.17 (1.06-1.30)	639	1.16 (1.08-1.26)
	Office administrative and other support activities	1641	1.09 (1.04-1.15)	1051	1.06 (0.99-1.12)	590	1.22 (1.12-1.32)
	Wholesale and retail trade	1554	1.05 (1.00-1.10)	745	1.04 (0.97-1.11)	809	1.07 (1.00-1.15)
	Construction	3702	1.04 (1.00-1.07)	2040	1.02 (0.98-1.07)	1662	1.08 (1.03-1.13)
Melanoma of skin	Electricity, gas, steam and air conditioning supply	112	1.28 (1.07-1.55)	11	1.04 (0.58-1.87)	101	1.21 (1.00-1.48)
	Financial, Insurance Activities And Business Support Service Activities	476	1.14 (1.04-1.25)	139	0.94 (0.79-1.12)	337	1.23 (1.10-1.38)

Connective and soft tissue	Manufacture of plastics products	35	1.43 (1.03-2.00)	13	1.42 (0.83-2.45)	22	1.40 (0.92-2.12)
Eye	Manufacture of electrical equipment	20	1.60 (1.03-2.49)	5	1.29 (0.54-3.11)	15	1.68 (1.01-2.81)
Brain	Railways	171	1.43 (1.24-1.66)	16	1.10 (0.68-1.79)	155	1.22 (1.05-1.43)
Other cancer sites	Manufacture of coke oven products	54	1.34 (1.04-1.72)	28	1.41 (1.00-2.00)	26	1.26 (0.88-1.80)
	Manufacture of pharmaceutical preparations	802	1.13 (1.06-1.21)	247	1.09 (0.97-1.23)	555	1.12 (1.03-1.21)
	Manufacture of refined petroleum products	631	1.11 (1.03-1.20)	188	1.02 (0.89-1.17)	443	1.12 (1.03-1.22)
	Railways	972	1.10 (1.04-1.17)	168	1.04 (0.90-1.20)	804	1.04 (0.98-1.11)
	Manufacture of paper and paper products	1153	1.07 (1.01-1.13)	457	1.06 (0.98-1.16)	696	1.05 (0.98-1.12)
	Manufacture of machinery, equipment and motor vehicles	18238	1.07 (1.05-1.08)	6855	1.03 (1.01-1.05)	11383	1.07 (1.06-1.09)
	Electricity, gas, steam and air conditioning supply	2034	1.07 (1.03-1.12)	255	0.93 (0.82-1.04)	1779	1.05 (1.00-1.09)
	Manufacture of wearing apparel	992	1.06 (1.00-1.13)	517	1.13 (1.04-1.23)	475	1.03 (0.94-1.12)
	Manufacture of basic chemicals	3541	1.06 (1.02-1.09)	1411	1.03 (0.98-1.08)	2130	1.06 (1.02-1.11)
	Wholesale and retail trade	9660	1.06 (1.04-1.08)	4284	1.05 (1.02-1.08)	5376	1.08 (1.05-1.11)
	Manufacture of wood products	3370	1.05 (1.01-1.08)	1436	1.01 (0.96-1.06)	1934	1.08 (1.04-1.13)
	Manufacture of basic metals	5062	1.04 (1.01-1.07)	1972	1.01 (0.97-1.05)	3090	1.04 (1.01-1.08)
	Printing	1512	1.04 (1.00-1.10)	525	1.00 (0.92-1.08)	987	1.05 (0.99-1.11)
	Manufacture of textiles	2819	1.04 (1.00-1.08)	1305	1.05 (1.00-1.11)	1514	1.03 (0.98-1.08)
	Manufacture of electrical equipment	3725	1.03 (1.00-1.06)	1392	1.00 (0.95-1.05)	2333	1.03 (0.99-1.07)
	Financial, Insurance Activities And Business Support Service Activities	6633	1.03 (1.01-1.05)	2058	0.96 (0.92-1.00)	4575	1.05 (1.03-1.08)
	Land transport	5544	1.03 (1.01-1.06)	1814	1.02 (0.98-1.07)	3730	1.01 (0.98-1.04)

(*) Abbreviations: N: deceased people employed in the sector with the longest duration of employment; PMR: proportional mortality ratio calculated on the longest period of employment; CI: 95% confidence interval; N_5: deceased people employed in the sector for less than 5 years; PMR_5: proportional mortality ratio calculated on the period of employment in the sector for less than 5 years; N_{10}: deceased people employed in the sector for more than 10 years; PMR_{10}: proportional mortality ratio calculated on the period of employment in the sector for more than 10 years.

Table S2 Occupational Mortality Matrix (OMM) based on proportional mortality ratios (PMRs) by disease and industrial sector - Males.

		Males					
**Cause of death**	**Industrial Sector**	**N**	**PMR**	**N_5**	**PMR_5**	**N_{10}**	**PMR_{10}**
Asbestosis	Building of ships and boats	48	27.07 (20.34-36.04)	25	23.91 (15.93-35.88)	23	31.64 (21.04-47.59)
	Manufacture of other products n.e.c.	32	8.94 (6.31-12.68)	30	11.85 (8.24-17.05)		
	Manufacture of concrete articles , cement and plaster	51	8.11 (6.13-10.71)	25	6.44 (4.31-9.64)	26	10.45 (7.10-15.38)

	Manufacture of refined petroleum products	9	6.58 (3.42-12.67)			7	10.99 (5.23-23.09)
	Water transport	8	4.36 (2.18-8.73)	5	3.30 (1.37-7.95)	3	6.24 (2.01-19.36)
	Railways	6	3.55 (1.59-7.89)			4	4.18 (1.56-11.18)
	Warehousing and storage, rental and leasing activities, travel agency	9	3.16 (1.64-6.08)	9	6.07 (3.15-11.70)		
	Mining and quarrying	6	2.24 (1.00-4.98)	4	2.13 (0.80-5.70)		
Silicosis	Mining and quarrying	240	13.42 (11.76-15.32)	175	11.11 (9.48-13.02)	65	16.91 (13.22-21.61)
	Manufacture of ceramic products	102	5.62 (4.61-6.86)	61	4.33 (3.34-5.60)	41	9.00 (6.61-12.27)
	Manufacture of glass and glass products	53	4.08 (3.11-5.34)	33	3.40 (2.41-4.80)	20	5.73 (3.69-8.89)
	Electricity, gas, steam and air conditioning supply	100	2.93 (2.40-3.58)	38	3.81 (2.75-5.26)	62	4.13 (3.19-5.35)
	Manufacture of concrete articles , cement and plaster	139	2.72 (2.29-3.23)	84	2.10 (1.68-2.63)	55	4.31 (3.29-5.64)
	Building of ships and boats	37	2.59 (1.87-3.58)	24	2.18 (1.46-3.27)	13	3.60 (2.09-6.20)
	Manufacture of basic metals	162	1.95 (1.66-2.28)	116	1.77 (1.46-2.14)	46	2.20 (1.65-2.94)
	Construction	485	1.49 (1.35-1.64)	373	1.28 (1.14-1.43)	112	1.53 (1.26-1.85)
Circulatory system diseases	Manufacture of tobacco products	145	1.23 (1.09-1.38)	70	1.10 (0.92-1.31)	75	1.40 (1.19-1.64)
	Agriculture	182573	1.08 (1.08-1.09)	50882	1.01 (1.00-1.02)	131691	1.17 (1.17-1.18)
Ischemic heart diseases	Repairing n.e.c.	120	1.18 (1.00-1.39)	72	1.07 (0.86-1.32)	48	1.35 (1.04-1.75)
	Manufacture of air and spacecraft and related machinery	516	1.09 (1.01-1.18)	167	1.11 (0.96-1.27)	349	1.08 (0.98-1.19)
	Accommodation	4016	1.05 (1.02-1.08)	2363	1.02 (0.98-1.06)	1653	1.08 (1.04-1.13)
	Office administrative and other support activities	13218	1.05 (1.03-1.06)	9004	1.02 (1.00-1.04)	4214	1.08 (1.05-1.11)
	Manufacture of paper and paper products	1614	1.05 (1.00-1.10)	765	1.05 (0.98-1.12)	849	1.04 (0.97-1.10)
	Printing	2058	1.05 (1.00-1.09)	858	1.01 (0.95-1.08)	1200	1.06 (1.00-1.12)
	Land transport	7435	1.05 (1.03-1.07)	2837	1.04 (1.01-1.08)	4598	1.05 (1.02-1.08)
	Financial, Insurance Activities And Business Support Service Activities	8677	1.03 (1.01-1.05)	3138	0.99 (0.96-1.03)	5539	1.06 (1.03-1.08)
	Wholesale and retail trade	12454	1.02 (1.00-1.03)	6492	1.03 (1.01-1.06)	5962	0.99 (0.96-1.01)
Cerebrovascular diseases	Manufacture of tobacco products	43	1.40 (1.06-1.84)	22	1.31 (0.89-1.92)	21	1.52 (1.02-2.26)
	Agriculture	48582	1.11 (1.10-1.12)	13507	1.04 (1.02-1.05)	35075	1.20 (1.19-1.22)
Respiratory system diseases	Mining and quarrying	1612	1.55 (1.48-1.62)	1119	1.70 (1.61-1.79)	493	1.27 (1.17-1.39)
Chronic obstructive pulmonary disease	Mining and quarrying	762	1.43 (1.34-1.53)	544	1.58 (1.46-1.71)	218	1.14 (1.00-1.29)
Nervous system diseases	Manufacture of pharmaceutical preparations	321	1.25 (1.12-1.39)	104	1.15 (0.95-1.38)	217	1.30 (1.14-1.48)
	Human health activities	209	1.14 (1.00-1.30)	61	0.88 (0.68-1.12)	148	1.29 (1.10-1.51)
	Manufacture of wearing apparel	370	1.13 (1.02-1.25)	196	1.13 (0.99-1.30)	174	1.15 (1.00-1.34)
	Manufacture of ceramic products	409	1.13 (1.02-1.24)	177	1.06 (0.92-1.23)	232	1.20 (1.06-1.36)

	Manufacture of rubber products	462	1.13 (1.03-1.24)	194	1.21 (1.06-1.39)	268	1.09 (0.97-1.23)
	Manufacture of plastics products	429	1.10 (1.01-1.21)	193	1.13 (0.98-1.30)	236	1.11 (0.98-1.25)
	Financial, Insurance Activities And Business Support Service Activities	2263	1.06 (1.02-1.11)	717	0.94 (0.88-1.01)	1546	1.13 (1.07-1.19)
	Manufacture of basic metals	1875	1.05 (1.00-1.10)	868	1.09 (1.02-1.16)	1007	1.04 (0.98-1.11)
	Manufacture of machinery, equipment and motor vehicles	6135	1.03 (1.00-1.06)	2684	1.05 (1.02-1.09)	3451	1.03 (1.00-1.06)
Mental disorders	Animal production	7	2.45 (1.20-4.99)	7	2.78 (1.37-5.68)		
Accidental falls	Construction	1798	1.22 (1.17-1.28)	969	1.10 (1.03-1.17)	829	1.36 (1.27-1.46)
Other diseases	Waste collection, treatment and disposal activities	752	1.20 (1.13-1.28)	380	1.20 (1.10-1.32)	372	1.19 (1.08-1.30)
	Water collection, treatment and supply	169	1.17 (1.02-1.35)	59	1.08 (0.85-1.36)	110	1.24 (1.05-1.47)
	Office administrative and other support activities	14827	1.14 (1.12-1.16)	10595	1.12 (1.10-1.14)	4232	1.08 (1.05-1.11)
	Accommodation	4941	1.13 (1.10-1.16)	3158	1.10 (1.06-1.13)	1783	1.09 (1.05-1.14)
	Human health activities	869	1.11 (1.04-1.18)	355	1.07 (0.97-1.18)	514	1.14 (1.05-1.23)
	Warehousing and storage, rental and leasing activities, travel agency	1967	1.10 (1.05-1.14)	926	1.19 (1.12-1.26)	1041	1.04 (0.98-1.10)
	Water transport	1253	1.09 (1.03-1.14)	834	1.09 (1.03-1.16)	419	1.02 (0.93-1.11)
	Financial, Insurance Activities And Business Support Service Activities	9455	1.03 (1.01-1.05)	3961	1.07 (1.04-1.10)	5494	1.00 (0.98-1.03)
	Agriculture	65064	1.02 (1.02-1.03)	19438	0.99 (0.98-1.01)	45626	1.07 (1.06-1.08)

(*) Abbreviations: N: deceased people employed in the sector with the longest duration of employment; PMR: proportional mortality ratio calculated on the longest period of employment; CI: 95% confidence interval; N_5: deceased people employed in the sector for less than 5 years; PMR_5: proportional mortality ratio calculated on the period of employment in the sector for less than 5 years; N_{10}: deceased people employed in the sector for more than 10 years; PMR_{10}: proportional mortality ratio calculated on the period of employment in the sector for more than 10 years.

Table S3 Occupational Mortality Matrix (OMM) based on proportional mortality ratios (PMRs) by malignant neoplasm and industrial sector - Females.

		**Females**					
**Cause of death**	**Industrial Sector**	**N**	**PMR**	**N_5**	**PMR_5**	**N_{10}**	**PMR_{10}**
All neoplasms	Manufacture of wearing apparel	11641	1.07 (1.05-1.08)	6478	1.01 (0.99-1.03)	5163	1.22 (1.19-1.24)
	Manufacture of electrical equipment	6010	1.07 (1.06-1.08)	2714	1.03 (1.02-1.04)	3296	1.18 (1.15-1.21)
	Manufacture of pharmaceutical preparations	1374	1.07 (1.03-1.12)	541	1.00 (0.93-1.06)	833	1.19 (1.13-1.26)
	Wholesale and retail trade	20249	1.06 (1.05-1.07)	10635	1.01 (1.00-1.02)	9614	1.22 (1.20-1.24)
	Manufacture of plastics products	2207	1.06 (1.03-1.10)	1194	1.04 (1.00-1.09)	1013	1.18 (1.12-1.23)
	Printing	1963	1.06 (1.02-1.09)	903	0.99 (0.95-1.04)	1060	1.20 (1.15-1.25)

	Manufacture of basic chemicals	2554	1.05 (1.02-1.08)	1289	0.99 (0.95-1.04)	1265	1.17 (1.12-1.22)
	Manufacture of textiles	12227	1.05 (1.03-1.06)	7238	1.01 (0.99-1.02)	4989	1.20 (1.17-1.22)
	Manufacture of machinery, equipment and motor vehicles	12059	1.04 (1.03-1.06)	5708	0.99 (0.97-1.01)	6351	1.16 (1.14-1.18)
	Manufacture of leather and related products	5197	1.04 (1.02-1.06)	2922	1.02 (0.99-1.04)	2275	1.11 (1.08-1.14)
	Office administrative and other support activities	34554	1.03 (1.02-1.04)	25277	1.05 (1.04-1.06)	9277	1.09 (1.08-1.11)
	Manufacture of wood products	2777	1.03 (1.00-1.06)	1567	0.99 (0.96-1.03)	1210	1.15 (1.10-1.20)
	Land transport	2068	1.03 (1.00-1.07)	871	0.98 (0.94-1.03)	1197	1.13 (1.08-1.18)
	Manufacture of jewellery	6	2.81 (1.26-6.26)			4	3.90 (1.46-10.41)
	Manufacture of rubber products	15	2.74 (1.65-4.53)	10	3.15 (1.70-5.83)	5	2.08 (0.86-4.99)
Mesothelioma	Manufacture of concrete articles , cement and plaster	11	2.39 (1.33-4.31)	5	1.73 (0.72-4.15)	6	3.39 (1.53-7.54)
	Manufacture of textiles	136	2.20 (1.86-2.60)	74	1.78 (1.42-2.24)	62	2.82 (2.18-3.65)
	Manufacture of basic metals	18	1.98 (1.25-3.13)	13	2.50 (1.45-4.29)	5	1.24 (0.52-2.98)
	Manufacture of basic chemicals	24	1.81 (1.22-2.70)	15	1.96 (1.18-3.24)	9	1.55 (0.81-2.98)
	Manufacture of machinery, equipment and motor vehicles	109	1.77 (1.46-2.14)	52	1.57 (1.19-2.06)	57	1.96 (1.50-2.57)
	Printing	17	1.70 (1.05-2.73)	9	1.71 (0.89-3.28)	8	1.65 (0.82-3.32)
	Manufacture of wood products	24	1.60 (1.07-2.39)	11	1.18 (0.65-2.13)	13	2.21 (1.28-3.83)
	Manufacture of electrical equipment	45	1.50 (1.12-2.01)	20	1.31 (0.84-2.02)	25	1.65 (1.11-2.46)
	Manufacture of wearing apparel	80	1.38 (1.10-1.72)	39	1.11 (0.81-1.52)	41	1.75 (1.27-2.41)
	Financial, Insurance Activities And Business Support Service Activities	105	1.30 (1.06-1.58)	47	1.05 (0.78-1.41)	58	1.63 (1.23-2.15)
	Wholesale and retail trade	127	1.25 (1.04-1.50)	63	1.06 (0.82-1.37)	64	1.51 (1.16-1.96)
Trachea, bronchus and lung	Manufacture of refined petroleum products	48	1.77 (1.35-2.31)	16	1.33 (0.82-2.13)	32	2.07 (1.50-2.87)
	Repairing n.e.c.	18	1.73 (1.11-2.69)	11	1.49 (0.84-2.65)	7	2.07 (1.04-4.12)
	Printing	353	1.56 (1.41-1.73)	167	1.41 (1.22-1.63)	186	1.70 (1.48-1.95)
	Wholesale and retail trade	3052	1.32 (1.27-1.37)	1588	1.16 (1.10-1.22)	1464	1.51 (1.43-1.59)
	Manufacture of basic chemicals	349	1.24 (1.12-1.37)	155	0.96 (0.82-1.12)	194	1.54 (1.34-1.76)
	Manufacture of other products n.e.c.	276	1.24 (1.11-1.39)	171	1.11 (0.96-1.29)	105	1.46 (1.22-1.76)
	Manufacture of pharmaceutical preparations	183	1.23 (1.07-1.42)	63	0.92 (0.72-1.17)	120	1.47 (1.24-1.75)
	Manufacture of electrical equipment	801	1.22 (1.14-1.31)	377	1.14 (1.03-1.25)	424	1.27 (1.16-1.40)
	Accommodation	1593	1.21 (1.15-1.27)	1136	1.10 (1.04-1.16)	457	1.59 (1.45-1.74)
	Financial, Insurance Activities And Business Support Service Activities	2307	1.21 (1.16-1.26)	1103	1.01 (0.96-1.08)	1204	1.46 (1.37-1.54)
	Manufacture of rubber products	140	1.21 (1.03-1.42)	68	1.03 (0.81-1.29)	72	1.39 (1.12-1.74)
	Land transport	294	1.20 (1.07-1.34)	112	0.96 (0.80-1.14)	182	1.39 (1.21-1.60)
	Manufacture of ceramic products	183	1.17 (1.02-1.34)	94	1.05 (0.86-1.27)	89	1.28 (1.05-1.57)
	Human health activities	753	1.16 (1.08-1.25)	322	0.98 (0.88-1.09)	431	1.34 (1.23-1.47)
	Office administrative and other support activities	4520	1.15 (1.12-1.18)	3213	1.06 (1.02-1.10)	1307	1.34 (1.27-1.42)
	Manufacture of machinery, equipment and motor vehicles	1588	1.15 (1.09-1.21)	735	0.99 (0.92-1.06)	853	1.30 (1.22-1.39)

	Manufacture of basic metals	223	1.15 (1.01-1.31)	111	1.01 (0.84-1.21)	112	1.30 (1.09-1.55)
	Manufacture of leather and related products	655	1.09 (1.01-1.18)	380	1.04 (0.94-1.15)	275	1.13 (1.01-1.27)
	Manufacture of wearing apparel	1367	1.06 (1.01-1.12)	756	0.96 (0.89-1.03)	611	1.17 (1.08-1.27)
Larynx	Manufacture of basic metals	7	2.24 (1.07-4.71)	3	1.57 (0.51-4.85)	4	3.08 (1.15-8.25)
	Accommodation	34	1.61 (1.14-2.28)	27	1.51 (1.02-2.24)	7	1.59 (0.75-3.38)
Pharynx	Accommodation	78	1.36 (1.07-1.72)	63	1.31 (1.01-1.71)	15	1.30 (0.78-2.19)
Ovary	Manufacture of air and spacecraft and related machinery	13	1.90 (1.13-3.20)	5	2.16 (0.95-4.91)	8	1.61 (0.82-3.14)
Colon-Rectum	Manufacture of refined petroleum products	38	1.59 (1.17-2.16)	14	1.35 (0.82-2.25)	24	1.67 (1.14-2.45)
	Washing and dry-cleaning of textile and fur products	162	1.18 (1.01-1.37)	112	1.25 (1.04-1.50)	50	1.05 (0.81-1.38)
	Manufacture of wearing apparel	1194	1.08 (1.02-1.15)	679	1.08 (1.00-1.16)	515	1.07 (0.98-1.16)
	Office administrative and other support activities	3663	1.03 (1.00-1.07)	2692	1.06 (1.02-1.10)	971	1.02 (0.96-1.09)
Liver	Manufacture of basic precious and non-ferrous metals	27	1.63 (1.12-2.36)	23	2.26 (1.51-3.37)	4	0.59 (0.22-1.58)
	Manufacture of electrical equipment	253	1.22 (1.08-1.37)	146	1.35 (1.15-1.59)	107	1.01 (0.84-1.23)
	Manufacture of basic chemicals	111	1.20 (1.00-1.44)	67	1.21 (0.96-1.54)	44	1.10 (0.82-1.48)
	Office administrative and other support activities	1320	1.07 (1.02-1.14)	973	1.03 (0.97-1.10)	347	1.10 (0.99-1.23)
Stomach	Manufacture of wood products	184	1.20 (1.04-1.38)	106	1.20 (1.00-1.45)	78	1.21 (0.97-1.50)
Kidney	Mining and quarrying	7	2.11 (1.01-4.40)	3	1.54 (0.50-4.74)	4	2.98 (1.13-7.84)
	Manufacture of wood products	63	1.36 (1.06-1.74)	35	1.30 (0.93-1.81)	28	1.48 (1.02-2.14)
	Manufacture of electrical equipment	113	1.22 (1.01-1.46)	49	1.10 (0.83-1.46)	64	1.31 (1.02-1.67)
	Manufacture of wearing apparel	213	1.19 (1.04-1.36)	108	1.06 (0.87-1.28)	105	1.39 (1.14-1.69)
	Manufacture of textiles	239	1.19 (1.05-1.35)	148	1.18 (1.01-1.39)	91	1.22 (0.99-1.50)
	Manufacture of machinery, equipment and motor vehicles	224	1.16 (1.02-1.33)	98	1.00 (0.82-1.22)	126	1.32 (1.11-1.58)
Bladder	Manufacture of refined petroleum products	6	2.27 (1.02-5.04)	3	2.32 (0.75-7.17)	3	2.17 (0.70-6.69)
	Printing	33	1.76 (1.25-2.47)	16	1.52 (0.93-2.48)	17	2.00 (1.24-3.22)
	Hairdressing saloons	13	1.75 (1.02-3.02)	10	1.67 (0.90-3.12)	3	2.07 (0.67-6.42)
	Manufacture of plastics products	34	1.50 (1.07-2.10)	15	1.03 (0.62-1.71)	19	2.23 (1.42-3.50)
	Wholesale and retail trade	248	1.39 (1.22-1.58)	143	1.31 (1.11-1.55)	105	1.45 (1.19-1.77)
	Manufacture of electrical equipment	82	1.38 (1.11-1.71)	47	1.43 (1.07-1.90)	35	1.28 (0.92-1.78)
	Accommodation	148	1.35 (1.15-1.58)	111	1.26 (1.04-1.52)	37	1.55 (1.12-2.14)
	Manufacture of textiles	171	1.25 (1.07-1.45)	112	1.12 (0.93-1.35)	59	1.43 (1.10-1.84)
	Financial, Insurance Activities And Business Support Service Activities	163	1.21 (1.03-1.41)	86	1.13 (0.91-1.40)	77	1.28 (1.02-1.61)
	Office administrative and other support activities	442	1.18 (1.07-1.29)	316	1.09 (0.97-1.22)	126	1.34 (1.13-1.60)
Melanoma of skin	Manufacture of refined petroleum products	8	2.27 (1.15-4.49)			7	3.12 (1.52-6.43)
Eye	Manufacture of basic chemicals	8	2.74 (1.37-5.50)	4	2.68 (1.00-7.14)	4	2.68 (0.99-7.22)
Other cancer sites	Building of ships and boats	53	1.34 (1.04-1.73)	34	1.52 (1.11-2.09)	19	1.09 (0.71-1.68)
	Manufacture of wearing apparel	2787	1.05 (1.01-1.09)	1557	1.01 (0.97-1.06)	1230	1.09 (1.03-1.15)
	Manufacture of electrical equipment	1445	1.05 (1.00-1.10)	678	1.02 (0.95-1.09)	767	1.06 (0.99-1.13)

	Manufacture of textiles	3075	1.05 (1.01-1.08)	1889	1.01 (0.97-1.05)	1186	1.08 (1.02-1.14)

(*) Abbreviations: N: deceased people employed in the sector with the longest duration of employment; PMR: proportional mortality ratio calculated on the longest period of employment; CI: 95% confidence interval; N_5: deceased people employed in the sector for less than 5 years; PMR_5: proportional mortality ratio calculated on the period of employment in the sector for less than 5 years; N_{10}: deceased people employed in the sector for more than 10 years; PMR_{10}: proportional mortality ratio calculated on the period of employment in the sector for more than 10 years.

Table S4 Occupational Mortality Matrix (OMM) based on proportional mortality ratios (PMRs) by disease and industrial sector - Females.

		**Females**					
**Cause of death**	**Industrial Sector**	**N**	**PMR**	**N_5**	**PMR_5**	**N_{10}**	**PMR_{10}**
Asbestosis	Manufacture of textiles	7	6.61 (3.10-14.11)	4	4.01 (1.47-10.90)	3	11.27 (3.14-40.40)
Silicosis	Manufacture of ceramic products	10	152.81 (77.31-302.06)	6	128.20 (48.90-336.10)	4	213.96 (78.36-584.22)
Circulatory system diseases	Agriculture	224422	1.17 (1.17-1.18)	73914	1.04 (1.03-1.04)	150508	1.43 (1.43-1.44)
Ischemic heart diseases	Animal production	14	1.70 (1.08-2.69)	13	1.60 (0.99-2.58)		
	Agriculture	62262	1.10 (1.09-1.11)	20957	1.00 (0.99-1.01)	41305	1.32 (1.31-1.34)
	Construction	1166	1.06 (1.01-1.12)	748	1.06 (0.99-1.13)	418	1.06 (0.97-1.16)
Cerebrovascular diseases	Agriculture	65608	1.16 (1.15-1.17)	21468	1.02 (1.01-1.04)	44140	1.43 (1.42-1.45)
Respiratory system diseases	Manufacture of rubber products	197	1.24 (1.09-1.42)	139	1.28 (1.09-1.50)	58	1.13 (0.88-1.46)
	Manufacture of machinery, equipment and motor vehicles	1663	1.09 (1.04-1.14)	1047	1.08 (1.02-1.14)	616	1.09 (1.01-1.17)
	Accommodation	1441	1.08 (1.03-1.13)	1148	1.03 (0.97-1.09)	293	1.15 (1.03-1.29)
	Financial, Insurance Activities And Business Support Service Activities	1429	1.06 (1.01-1.11)	884	1.07 (1.01-1.14)	545	1.02 (0.94-1.11)
Chronic obstructive pulmonary disease	Accommodation	596	1.21 (1.11-1.31)	480	1.13 (1.03-1.23)	116	1.27 (1.06-1.52)
	Manufacture of plastics products	126	1.21 (1.02-1.44)	85	1.12 (0.91-1.39)	41	1.31 (0.96-1.77)
	Manufacture of machinery, equipment and motor vehicles	668	1.18 (1.10-1.27)	416	1.12 (1.02-1.23)	252	1.24 (1.10-1.40)
	Financial, Insurance Activities And Business Support Service Activities	553	1.12 (1.03-1.21)	325	1.04 (0.94-1.16)	228	1.19 (1.05-1.36)
	Office administrative and other support activities	1945	1.06 (1.01-1.11)	1481	1.00 (0.95-1.05)	464	1.09 (1.00-1.20)
Nervous system diseases	Waste collection, treatment and disposal activities	35	1.65 (1.20-2.27)	23	1.43 (0.97-2.13)	12	2.27 (1.32-3.88)
	Land transport	240	1.18 (1.04-1.33)	120	1.16 (0.97-1.38)	120	1.20 (1.01-1.43)
	Manufacture of basic chemicals	342	1.16 (1.04-1.28)	216	1.15 (1.01-1.30)	126	1.16 (0.97-1.37)
	Manufacture of textiles	1651	1.14 (1.09-1.20)	1197	1.14 (1.08-1.21)	454	1.11 (1.01-1.21)

	Manufacture of wearing apparel	1188	1.11 (1.05-1.18)	785	1.12 (1.05-1.20)	403	1.08 (0.98-1.19)
	Manufacture of leather and related products	593	1.11 (1.03-1.20)	378	1.08 (0.98-1.20)	215	1.14 (1.00-1.30)
	Office administrative and other support activities	4095	1.05 (1.02-1.08)	2991	1.00 (0.96-1.03)	1104	1.15 (1.09-1.22)
	Manufacture of machinery, equipment and motor vehicles	1311	1.05 (1.00-1.11)	765	1.04 (0.97-1.12)	546	1.05 (0.96-1.14)
External causes of morbidity	Retail sale of automotive fuel (petrol stations)	19	1.65 (1.08-2.53)	13	1.35 (0.81-2.23)	6	2.18 (1.00-4.73)
	Hairdressing saloons	126	1.34 (1.13-1.58)	108	1.13 (0.95-1.35)	18	1.42 (0.91-2.22)
	Accommodation	1170	1.16 (1.09-1.22)	992	1.13 (1.06-1.20)	178	0.92 (0.80-1.06)
Accidental falls	Manufacture of pharmaceutical preparations	31	1.69 (1.19-2.40)	15	1.52 (0.92-2.52)	16	1.90 (1.17-3.09)
	Electricity, gas, steam and air conditioning supply	18	1.62 (1.02-2.57)			18	2.24 (1.41-3.54)
	Manufacture of leather and related products	88	1.39 (1.13-1.72)	55	1.33 (1.02-1.73)	33	1.51 (1.08-2.13)
	Manufacture of electrical equipment	94	1.31 (1.07-1.61)	56	1.39 (1.07-1.80)	38	1.22 (0.89-1.68)
	Accommodation	157	1.19 (1.02-1.40)	128	1.23 (1.04-1.46)	29	1.04 (0.73-1.50)
Other diseases	Agriculture	83385	1.11 (1.11-1.12)	29977	1.09 (1.08-1.10)	53408	1.22 (1.21-1.23)
	Manufacture of food products	2651	1.04 (1.01-1.08)	1924	1.06 (1.02-1.10)	727	0.97 (0.90-1.03)

(*) Abbreviations: N: deceased people employed in the sector with the longest duration of employment; PMR: proportional mortality ratio calculated on the longest period of employment; CI: 95% confidence interval; N_5: deceased people employed in the sector for less than 5 years; PMR_5: proportional mortality ratio calculated on the period of employment in the sector for less than 5 years; N_{10}: deceased people employed in the sector for more than 10 years; PMR_{10}: proportional mortality ratio calculated on the period of employment in the sector for more than 10 years.
